# Extracellular Matrix From Decellularized Wharton’s Jelly Improves the Behavior of Cells From Degenerated Intervertebral Disc

**DOI:** 10.3389/fbioe.2020.00262

**Published:** 2020-03-27

**Authors:** Letizia Penolazzi, Michela Pozzobon, Leticia Scussel Bergamin, Stefania D’Agostino, Riccardo Francescato, Gloria Bonaccorsi, Pasquale De Bonis, Michele Cavallo, Elisabetta Lambertini, Roberta Piva

**Affiliations:** ^1^Department of Biomedical and Specialty Surgical Sciences, University of Ferrara, Ferrara, Italy; ^2^Stem Cells and Regenerative Medicine Lab, Fondazione Istituto di Ricerca Pediatrica Città della Speranza, Padua, Italy; ^3^Department of Women and Children Health, University of Padova, Padua, Italy; ^4^Section of Obstetrics and Gynecology, Department of Morphology, Surgery and Experimental Medicine, University of Ferrara, and S. Anna University Hospital, Ferrara, Italy; ^5^Department of Neurosurgery, University of Ferrara, and S. Anna University Hospital, Ferrara, Italy

**Keywords:** Wharton’s jelly, intervertebral disc cells, TRPS1, decellularized matrix, scaffold

## Abstract

Regenerative therapies for intervertebral disc (IVD) injuries are currently a major challenge that is addressed in different ways by scientists working in this field. Extracellular matrix (ECM) deriving from decellularized non-autologous tissues has been established as a biomaterial with remarkable regenerative capacity and its potential as a therapeutic agent is rising. In the present study, we investigated the potential of decellularized Wharton’s jelly matrix (DWJM) from human umbilical cord to act as an ECM-based scaffold for IVD cell culturing. An efficient detergent-enzymatic treatment (DET) was used to produce DWJM maintaining its native microarchitecture. Afterward, immunofluorescence, biochemical assays and electron microscopy analysis showed that DWJM was able to produce sizeable 3D cell aggregates, when combined with human mesenchymal stromal cells isolated from WJ (MSCs) and IVD cells. These latter cells are characterized by the loss of their chondrocyte-like phenotype since they have been isolated from degenerated IVD and *in vitro* expanded to further de-differentiate. While the effect exerted by DWJM on MSCs was essentially the induction of proliferation, conversely, on IVD cells the DWJM promoted cell differentiation toward a discogenic phenotype. Notably, for the first time, the ability of DWJM to improve the degenerated phenotype of human IVD cells was demonstrated, showing that the mere presence of the matrix maintained the viability of the cells, and positively affected the expression of critical regulators of IVD homeostasis, such as SOX2, SOX9, and TRPS1 transcription factors at specific culture time. Our data are in line with the hypothesis that the strengthening of cell properties in terms of viability and expression of specific proteins at precise times represents an important condition in the perspective of guiding the recovery of cellular functionality and triggering regenerative potential. Currently, there are no definitive surgical or pharmacological treatments for IVD degeneration (IDD) able to restore the disc structure and function. Therefore, the potential of DWJM to revert degenerated IVD cells could be exploited in the next future an ECM-based intradiscal injectable therapeutic.

## Introduction

The intervertebral disc (IVD) is a natural composite system bonding two adjacent vertebrae. IVD degeneration (IDD) is a phenomenon that can occur as a result of the natural aging, trauma or pathological conditions, and represents one of the main causes of lower back pain that, when it becomes chronic and debilitating, has a serious socio-economic impact (Roughley, 2004; Murray et al., 2012; Geurts et al., 2018; Fujii et al., 2019). To date, spinal fusion surgery is the treatment of choice for IDD aimed at removing the painful symptoms and the degenerated part of the disc (Amin et al., 2017). However, the surgical approach cannot slow down the degenerative process or restore the normal mechanical functionality of the disc, and management of chronic low back pain remains complicated (Schizas et al., 2010; Ohtori et al., 2011). In the recent years, several tissue engineering strategies have been developed and proposed as potential innovative approaches in IDD therapeutic treatment. In all its anatomical and functional complexity starting from the nucleus pulposus (NP, highly hydrophilic, rich of proteoglycans and collagen, with shock absorbing function), up to the annulus (AF, lamellar fibrocartilaginous tissue, with containment function) and cartilaginous endplates (a more rigid tissue acting as interface between disc and vertebrae), the IVD is attracting considerable interest from the scientists involved in tissue engineering field (Buckley et al., 2018; Farrugia et al., 2019; Tendulkar et al., 2019). In particular, several studies aim at developing innovative bio-inspired scaffolds, in order to counteract extracellular matrix (ECM) loss following degeneration/inflammation, and support functional recovery by endogenous damaged microenvironment (Coogan et al., 2016; Van Uden et al., 2017; Gullbrand et al., 2018). In this scenario, decellularized ECM deriving from autologous or non-autologous tissues represents an important advance in this field, as ideal system to deliver chemokines and growth factors, and provides adequate biomechanical microarchitecture also in the damaged IVD microenvironment (D’Este et al., 2018; Hensley et al., 2018; Liu et al., 2019; Ventre et al., 2019; Zhao et al., 2019). Obviously, obtaining autologous ECM from human IVD is not feasible, but recent evidences based on decellularized ECM from IVD of animal origin are encouraging (Illien-Jünger et al., 2016; Lin et al., 2016; Zhou et al., 2018; Xu et al., 2019). However, these scaffolds, which are potentially implantable in humans, can have non-negligibile side effects because of immunological reactions and pathogen transmission risk (Zhou et al., 2018). Interesting alternatives can come from tissues for which obtaining the ECM can be easily achieved, both from a technical and ethical point of view. In these terms, the perinatal tissues may represent a valuable opportunity.

In the current study, we produced decellularized ECM obtained from human Wharton’s jelly (WJ) of full term human umbilical cord, and investigated its potential as bio-inspired scaffold affecting the behavior of IVD cells. WJ is a mucous connective tissue that surrounds the umbilical cord vessels and is covered by a layer of simple amniotic epithelium (Ferguson and Dodson, 2009). The recent characterization of WJ suggests that its composition is particularly similar to that of IVD. In particular, the abundance of hyaluronic acid (HA) makes this tissue strongly hydrated, viscous and suitable as potential substitute of NP (Jadalannagari et al., 2017; Bullard et al., 2019). In addition, WJ, being rich in growth factors and cytokines (Penolazzi et al., 2010; Jadalannagari et al., 2017; Najar et al., 2018; Kehtari et al., 2019) is particularly attractive for potential application in tissue engineering (Beiki et al., 2017; Basiri et al., 2019; Li et al., 2019; Walker et al., 2019). In recent years, different methods to decellularize the WJ have been developed and compared, aimed at producing a scaffold able to sustain cell viability and proliferation, and preserve the native ECM microstructure and secretion profile (Stocco et al., 2014; Beiki et al., 2017; Jadalannagari et al., 2017).

Here, we produced decellularized Wharton’s jelly matrix (DWJM) with an efficient method based on detergent-enzymatic treatment (DET) cycle (Piccoli et al., 2016). The ability of DWJM to generate sizeable 3D cell aggregates was investigated by using human cells from degenerated IVD as cellular model of disease, and human mesenchymal stromal cells isolated from WJ (MSCs) as control cellular model. For the first time, we demonstrated the ability of DWJM to improve the degenerated phenotype of IVD cells isolated from herniated lumbar disc, expanded and further de-differentiated during the passage in culture. When combined with DWJM, the IVD cells were viable and recovered their chondrogenic-like phenotype through the expression of important proteins such as SOX2, SOX9, and TRPS1, previously recognized as critical regulators of IVD homeostasis (Penolazzi et al., 2019).

Therefore, we proposed DWJM as innovative scaffold mimicking the ECM of IVD microenvironment, and able to restore the chondrocyte-like cell phenotype.

## Materials and Methods

### Cells

Human umbilical cords (all from natural deliveries, *n* = 5) were collected after mothers’ consent and approval of the Ethics Committee of the University of Ferrara and S. Anna Hospital (protocol approved on November 19th, 2006). Harvesting procedures of WJ from umbilical cord were conducted in full accordance with the Declaration of Helsinki as adopted by the 18th World Medical Assembly in 1964 and successively revised in Edinburgh (2000) and the Good Clinical Practice guidelines. Cords were processed within 4 h and stored in sterile saline until use (Penolazzi et al., 2012). Typically, the cord was rinsed several times with sterile phosphate-buffered saline (PBS) before processing and was cut into pieces (2–4 cm in length). Single pieces were dissected, after separating the epithelium of each section along its length, to expose the underlying WJ. The soft gel tissue was then finely chopped. The same tissue (2–3 mm^2^ pieces) was placed directly into a 25 cm^2^ flask for culture expansion in 10% Fetal Calf Serum (Euroclone S.p.A., Milan, Italy) Dulbecco’s Modified Eagle’s Medium (DMEM) low-glucose supplemented with antibiotics (100 μg/mL streptomycin, 100 U/mL penicillin), at 37°C in a humidified atmosphere of 5% CO_2_. After 5–7 days, the culture medium was removed and then changed twice a week. At 70–80% confluence, cells were scraped off by 0.05% trypsin-ethylenediaminetetraaceticacid (EDTA) (Sigma Aldrich, St. Louis, United States) washed, counted by hemocytometric analysis, assayed for viability, and used thereafter for *in vitro* experiments (passages P2–P3).

Surgical herniated human disc tissues were obtained from six patients (patients’ age was between 31 and 77 years, mean age 59 years, three males and three females, Pfirrmann grade 3–4), using research protocol approved by Ethics Committee of the University of Ferrara and S. Anna Hospital (protocol approved on November 17th, 2016). Patients were operated for the herniated lumbar disc through a microsurgical posterior approach. Disc sampling was obtained from the central core of the disc, in order to avoid anterior and posterior longitudinal ligament, annulus and calcified portion of the disc. Lumbar intervertebral disc tissues (1–2 cm^3^) were collected, cut into small pieces, and subjected to mild digestion in 15 mL centrifuge tube with only 1 mg/mL type IV collagenase (Sigma-Aldrich, St. Louis, United States) for 5 h at 37°C in DMEM high glucose/F12 (Euroclone S.p.A., Milan, Italy) as previously described (Penolazzi et al., 2019). After digestion, cell suspension was filtered with a Falcon^TM^ 70 μm Nylon Cell strainer (BD Biosciences, Franklin Lakes, NJ, United States). Subsequently 300 × *g* centrifugation was conducted for 10 min, the supernatant discarded, the cells resuspended in basal medium (DMEM/F12 containing 10% fetal calf serum, 100 μg/mL streptomycin, 100 U/mL penicillin, and 1% Glutamine) (Euroclone) and seeded in polystyrene culture plates (Sarstedt, Nümbrecht, Germany) at 10000 cells/cm^2^. The cells were maintained in culture at 37°C in a humidified atmosphere with 5% CO_2_. P0 cells were expanded by growing for a period not exceeding a week until subconfluent, detaching by trypsinization, and maintained in culture for more than two passages to obtain IVD cells that were used for later experiments. As already demonstrated after monolayer culture expansion, cells isolated from human lumbar IVD become de-differentiated, lose their chondrogenic-like phenotype resembling the degeneration process (Penolazzi et al., 2018).

### Decellularization

Several fragments of WJ were treated up to three cycles of the DET as previously described (Piccoli et al., 2016). Briefly: samples were washed two times in PBS and then placed in deionized water with 3% Penicillin/streptomicyn. For each DET cycle, samples were gently shacked in deionized water at 4°C for 24 h, then in 4% sodium deoxycholate (SDC-Sigma-Aldrich) at room temperature (RT) for 4 h, and finally in 2000 kU DNase-I (Sigma-Aldrich) in 1 M NaCl at RT for 3 h. (One cycle is formed by water/SDC/DNAse). After each cycles, samples underwent DNA extraction (Trizol, Invitrogen) and DNA quantification (Nanodrop). Decellularized samples were further dehydrated and used as scaffold for subsequent experiments.

### DWJM Seeding and Culture

Decellularized Wharton’s jelly matrix fragments (2–4 mg) were presoaked with DMEM high glucose for 1 h, seeded with 200–400 × 10^3^ cells and cultured in polypropylene tubes up to 14 days. Culture medium (DMEM high glucose/F12 or DMEM low glucose with 10% FCS) was added to cover the entire scaffold and changed twice a week. Each experiment refers to a single sample and the cells from the same donor are characterized before and after seeding.

### Biochemical Assay

The collagen content was quantified using a commercially available assay kit (Hydroxyproline Assay Kit, BioVision, Milpitas, CA, United States). The proteoglycan content was determined by measuring the amount of sulfated glycosaminoglycans (sGAG) in the papain-digested samples (Fresh WJ and DWJM) using the 1,9-dimethylmethylene blue (DMMB, Sigma-Aldrich) dye binding assay and spectrophotometry (Farndale et al., 1986). Samples were first lyophilized (dry weight, 30 mg) and then digested in papain buffer (250 μL papain in PBS at pH 6.0 with 150 mM sodium chloride, 55 nM sodium citrate, 5 mM cysteine-HCl, and 5 mM Na_2_EDTA) at 60°C for 12 h. After digestion, the supernatant fluid was incubated with DMMB and measured at 530 nm by a Microplate Absorbance Reader (Sunrise^TM^, Tecan, Männedorf-Switzerland).

### DNA Quantification

The DNA was extracted from both native and decellularized WJ. Six WJ specimens (wet weight, 25 mg) were used. The DNA was extracted using the DNA isolation kit (ReliaPrep gDNA Tissue Miniprep System, Promega, Fitchburg, WI, United States) and then quantified according to standard protocols by measuring the absorbance at 260/280 nm using a spectrophotometer (NanoDrop ND 1000; Thermo Scientific, Waltham, MA, United States).

### Histology and Immunofluorescence

Histological analyses were performed on frozen sections (7–10 μm thick) using haematoxylin and eosin (H&E) kit for rapid frozen section and Alcian Blue (AB) for glycosaminoglycan hematoxylin staining (all from Bio-Optica, Milan, Italy) under manufacturer’s instruction. For immunofluorescence analyses, frozen sections were permeabilized with 0.5% Triton X-100 (Sigma-Aldrich) in PBS for 10 min at RT, saturated with 10% horse serum (HS) (Thermo Fisher Scientific, WA, United States) in PBS for 12 min and with mouse serum (Sigma-Aldrich, dilution 1:10) for 15 min at RT. The samples were incubated with primary antibodies (Supplementary Table S1) for 1 h at 37°C and then with labeled secondary antibodies (Supplementary Table S1) for 1 h at 37°C. Nuclei were counterstained with fluorescent mounting medium plus 100 ng/ml 4′,6-diamidino-2-phenylindole (DAPI) (Sigma-Aldrich). Images were captured using an DMI6000B confocal microscope (Leica Microsystems, Wetzlar, Germany). The cell count percentage has been performed comparing the positive cells with all the nuclei present in the field (the number multiplied per 100). Percentage of seeded area, defined as DAPI fluorescence in all the seeded area (panoramic picture of the whole sections was made), and laminin and fibronectin mean fluorescence intensity has been calculated using Fiji 30 software as already performed elsewhere (Kim et al., 2016; Quarta et al., 2017, 2018) using 8 bit image and threshold (*n* = 6, 10 fields per each sample). All counts were made by two blinded operators.

### Electron Microscopy

The analysis of the morphological architecture, the porous structure, and the adhesion/integration of MSCs and IVD cells to DWJM was performed by different microscopic techniques, namely: inverted optical microscopy (Nikon Diafophot, Tokyo, Japan), scanning electron microscopy (SEM; Cambridge S360 microscope; Cambridge Instruments, Cambridge, United Kingdom), and transmission electron microscopy (TEM; ZEISS EM 910 electron microscope; Zeiss, Oberkochen, Germany). Samples for the electron microscopy, were fixed in glutaraldehyde 2.5% buffered solution and osmium tetroxide 2% buffered solution and dehydrated; for SEM analysis, samples were gold coated (Edward Sputter S150), while for TEM analysis, samples were araldite embedded (ACM Fluka Sigma-Aldrich) and the ultra-thin sections of a selected area were contrasted with uranyl acetate lead citrate. The pore size was estimated using SEM images, counting a minimum of 100 pores from different places on the cross section of the scaffolds. The pore sizes were analyzed by using Fiji 30 software.

### Proliferation and Viability

Viability assay was performed as previously described (Penolazzi et al., 2010). For Propidium Iodide and Calcein-AM analysis, cells were visualized under a fluorescence microscope (Nikon, Optiphot-2, Nikon corporation, Japan) using the filter block for fluorescein. Dead cells were stained in red, whereas viable ones appeared in green. The proliferation rate of MSCs and IVD cells was determined by using the alamarBlue^TM^ assay (Invitrogen Corporation, Carlsbad, CA, United States). The test is based on the metabolic activity of proliferating cells that results in a chemical reduction of alamarBlue^TM^ reagent, previously added to the *in vitro* cultured cells. Briefly, at sequential time points a medium containing 5% alamarBlue^TM^ was added to the cells, at 37°C and 5% CO_2_. After 4 h of incubation, 200 μL samples of culture medium were withdrawn, centrifuged, and subsequently placed on 96-well plates. Visible light absorption of the collected samples was determined at 570 and 620 nm by a Microplate Absorbance Reader (Sunrise^TM^, Tecan). Final values were calculated as the difference in absorbance units between the reduced and oxidized forms of alamarBlue^TM^.

### Statistical Analysis

Image-based counts and measurements were performed with Fiji 30 software (Mayachitra, Santa Barbara, CA, United States). For each analysis, at least five random pictures were used for data output. All graphs displayed were produced with GraphPad software 5 or 6 (GraphPad Software Inc., CA, United States). Data are expressed as means ± SD. Statistical significance was analyzed by unpaired Student’s *t*-test. *P*-value below 0.05 was considered to be statistically significant.

## Results

### Preparation and Characterization of Decellularized Human Wharton’s Jelly Matrix

**FIGURE 1:**
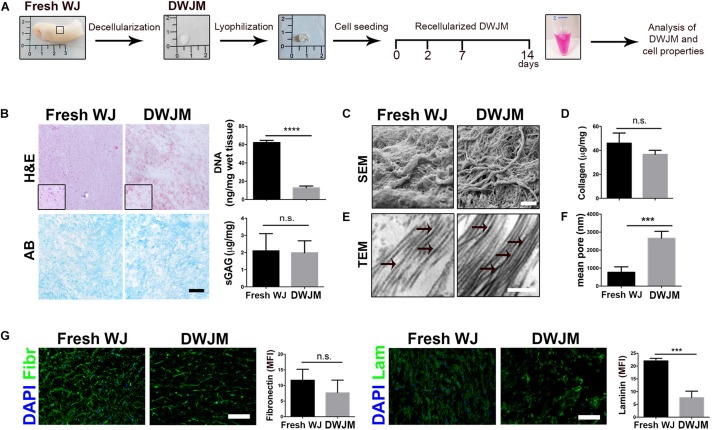
Preparation and characterization of decellularized human Wharton’s jelly matrix (DWJM). **(A)** Scheme of the different steps of the experimental plan: the gross appearance of Wharton’s jelly freshly isolated (Fresh WJ), Wharton’s jelly after decellularization (DWJM) and lyophilization process has been reported. After seeding of IVD cells or MSCs cells, the recellularized DWJM was kept in culture up to 14 days and then investigated. **(B)** Representative images of the histological analysis performed on Fresh WJ and DWJM by hematoxylin-eosin (H&E) and Alcian Blue (AB) staining. In the graphs the quantification of the residual DNA and total sulfated proteoglycans and glycosaminoglycans (sGAG) content is also reported. ****p < 0.0001; n.s., non-significant. Bar: 50 μm. **(C)** Representative images of SEM analysis of Fresh WJ and DWJM. Bar: 5 μm. **(D)** The quantification of the total collagen content (μg/mg of tissue) is reported in the graph. n.s., non-significant. **(E)** Representative images of TEM analysis of Fresh WJ and DWJM. Bar: 200 nm; black arrows indicate collagen fibers with visible cross-striation pattern. **(F)** Mean diameter of the pores (nm) is reported in the graph. ***p < 0.001; 10 analyzed fields, *n* = 3. **(G)** Representative images of the immunofluorescence analysis of Fibronectin (Fibr, in green), and Laminin (Lam, green) expression performed on Fresh WJ and DWJM. Nuclei were counterstained with DAPI (in blue); the results are expressed as mean fluorescence intensity (MFI, arbitrary unit) evaluated for 10 fields, *n* = 3. ***p < 0.001; n.s., non-significant. Bars: 50 μm.

The overview of the different steps of the experiment plan is schematically shown in Figure 1A. First, WJ from umbilical cord (fresh WJ) was decellularized by using a protocol based on DET, as already reported (Piccoli et al., 2016). Different WJ samples were subjected to one or more cycles of treatment in order to obtain DWJM by a complete cell removal. After each cycle, the gross appearance of treated tissues, DNA content and the ECM composition (laminin and fibronectin fluorescence) were analyzed demonstrating that one DET cycle was sufficient to achieve more than 95% of nuclei depletion, keeping the morphology of the sample (Figure 1B and Supplementary Figure S1). As shown in Figure 1B, the amount of DNA was below the value of 50 ng/mg of tissue which is the threshold concentration of residual DNA to avoid adverse host responses (Crapo et al., 2011). Preservation of native tissue architecture was investigated by sGAG content evaluation and electron microscopy analysis. The structure of the DWJM retained sGAG (Figure 1B) and collagen network (Figure 1C) similar to fresh WJ. In particular, SEM imaging revealed the maintenance of the ECM fibrillary microstructure after decellularization, together with the presence of irregular spaces between the fibrils that range from 0.5 to several microns (Figure 1C). Collagen quantification of the fresh and decellularized samples was not significantly different (41.5 ± 2.1 μg/mg for fresh WJ and 39.8 ± 0.65 μg/mg for the DWJM-Figure 1D). TEM showed the absence of intact cells and the presence of collagen fibers mainly running in the same orientation (Figure 1E). Accordingly, after decellularization, pore size modification was found. In particular, DWJM showed a significant increase in pore diameter (Figure 1F), which is a desired effect in order to facilitate cellular infiltration and subsequent recellularization of the decellularized matrix used as a scaffold.

Other primary ECM components such as non-collagenous fibronectin and laminin glycoproteins which participate in cell proliferation, adhesion, migration, differentiation activities, and reparative ECM role, were investigated in DWJM. As reported in Figure 1G, substantial levels of fibronectin and laminin were detected by fluorescent immunostaining, after decellularization, even if laminin content appeared to be less preserved.

As a whole, the decellularization process was effective in generating a cell-free porous matrix with its native microarchitecture and composition.

### Properties of the Cells Seeded on Decellularized Wharton’s Jelly Matrix

**FIGURE 2:**
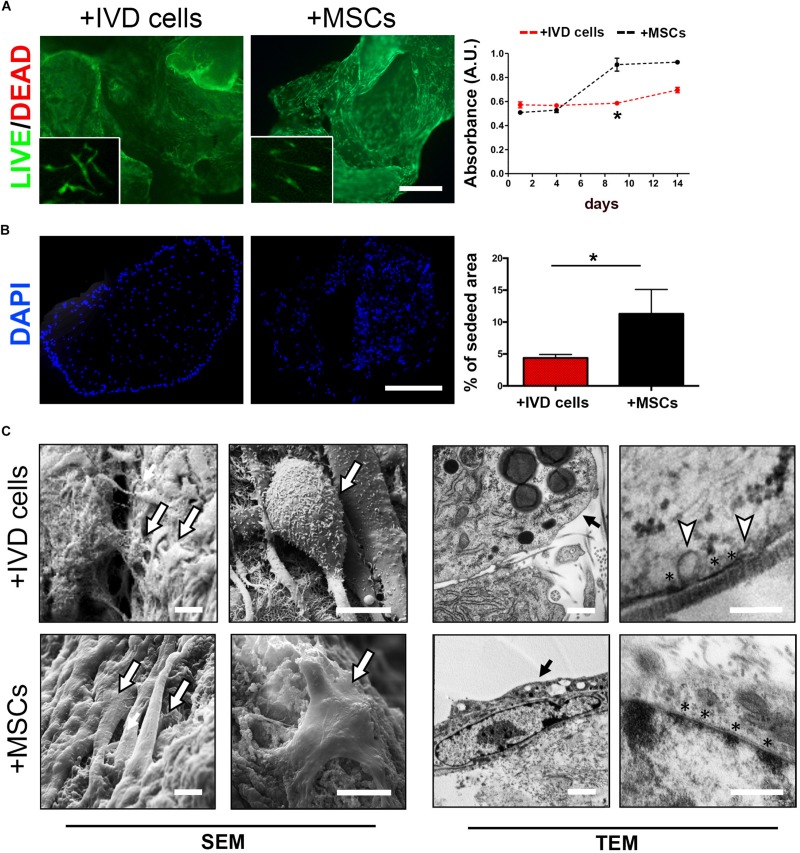
DWJM combined with chondrocyte – like IVD cells or mesenchymal stromal cells (MSCs) from WJ. **(A)** Effect on cell viability was determined by Calcein-AM/Propidium Iodide double staining after 7 days of culture. Dead cells (red cells) were undetectable. High magnification images are shown in the insets. Effect on cell proliferation was determined by alamarBlue^TM^ assay in IVD cells (red dotted lane) and MSCs (black dotted lane) cultured up to 14 days, data are presented as mean absorbance (A.U., absorbance unit ± SD, *n* = 3). **(B)** Effect on cell distribution was determined fluorescence microscopy analysis of DAPI-stained cells cultured up to 14 days. The percentage of seeded area is reported in the graph, *n* = 6. *p < 0.05. Bar: 200 μm. **(C)** Representative images of SEM and TEM analysis. The presence of seeded cells on the DWJM surface is indicated by the arrows. Asterisks (*) indicate electron-dense areas close to the cellular membrane, white triangles indicate the presence of vesicles budding from the cell surface. Bars: 2 μm (SEM images); 200 nm (TEM images).

In order to investigate the ability of DWJM to act as a scaffold for intervertebral disc cell culturing, human cells from degenerated intervertebral disc (namely IVD cells) were combined with the matrix and cultured in polypropylene tubes up to 14 days without soluble differentiation inducers. The effect of DWJM on IVD cells was compared with that on human MSCs from WJ, in terms of viability, proliferation and matrix interaction. By Calcein AM/Propidium Iodide staining, it was appreciable the ability of the cells to uniformly adhere to DWJM. As shown in Figure 2A, the cells were viable and able to maintain their spindle shape morphology. No major difference was observed when comparing the viability of both cell sources after 7 days of culture. However, alamarBlue^TM^ assay highlighted that growth rate of IVD cells was significantly slower compare to MSCs (Figure 2A), although the proliferation ability of these two cell types is absolutely comparable in 2D culture conditions (Penolazzi et al., 2018 and data not shown).

This was confirmed by fluorescence microscopy analysis of DAPI-stained cells which revealed a lower percentage of cell*-*seeding area in DWJM combined with IVD cells than in DWJM combined with MSCs (Figure 2B).

Scanning and transmission electron microscopic analysis clearly revealed the ability of the cells to create cell-cell and cell-matrix interconnections (Figure 2C). Electron-dense areas close to the cellular membrane as specific points of contact with matrix surface were appreciable. Intriguingly, abundant vesicles budding from the IVD cells surface were found. In particular, TEM analysis revealed that these vesicles, 100–200 nm in size (117.18 ± 20 nm, mean diameter), were distributed all over the cellular membrane.

### The Effect of DWJM on Degenerated Intervertebral Disc Cells

**FIGURE 3:**
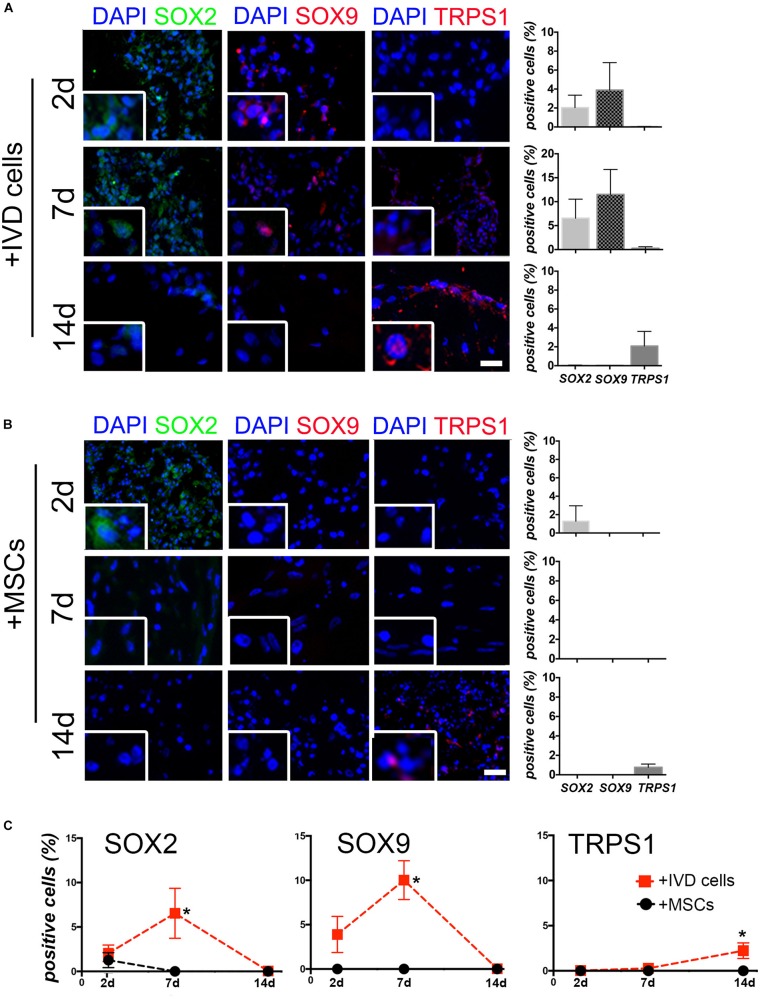
The effect of DWJM on degenerated IVD cells. Representative images of the immunofluorescence analysis of SOX2, SOX9, and TRPS1 expression performed on DWJM combined with IVD cells **(A)** or with MSCs **(B)** after 2d, 7d, and 14d (days) of culture. Nuclei were counterstained with DAPI (in blue); bars: 50 μm. High magnification images are shown in the insets. The percentage of positive cells (±SD) for each transcription factor is reported in the graphs (10 analyzed fields, *n* = 6). **(C)** Lines plot showing the average percentage (±SD) of SOX2, SOX9, and TRPS1 positive cells over the time are reported. *p < 0.05. Statistical analysis was performed for DWJM combined with IVD cells versus MSCs.

In a next step, we explored the potential of DWJM to restore the chondrocyte-like phenotype that belongs to healthy IVD cells and that is lost during degeneration/de-differentiation process. In particular, we evaluated the expression of three transcription factors that are lost in degenerated IVD microenvironment: SOX2, a well known stemness regulator, SOX9, the primary driver during the early stages of chondrogenic differentiation, and TRPS1, recently identified as pro-discogenic factor. As previously demonstrated (Penolazzi et al., 2019), the expression of these proteins always decreases during the passages in culture, as the cells undergo de-differentiation. Interestingly, as shown in Figure 3A and Supplementary Figures S2–S4, immunofluorescence analysis revealed that culturing degenerated IVD cells on DWJM up-regulated the expression of these transcription factors with a specific timing that resembles the early and late phases of discogenic/chondrogenic differentiation. In fact, a progressive increase of SOX2 and SOX9 expression levels was observed in the first seven days of culture, suggesting a cellular activity both in terms of stemness and differentiation supported by the presence of DWJM. In a second phase, between day 7 and day 14, a down-regulation of SOX2 and SOX9 together with an up-regulation of TRPS1 was observed, suggesting the achievement of a more mature cellular phenotype.

On the contrary, culturing MSCs on DWJM weakly affected the expression of the transcription factors analyzed (Figure 3B). Figure 3C well summarizes the switch on and off of the proteins, according to the cell type.

## Discussion

It has been repeatedly shown that there is no effective treatment protecting the IVD from degeneration. In the regenerative approaches in IVD tissue engineering aimed at restoring the AF and NP function, important results are expected from the development of specific biomimetic scaffolds. In the present study, we investigated the potential of DWJM as ECM-based scaffold able to provide appropriate physical and chemical milieu for IVD cells. For the first time, we demonstrated the ability of DWJM to improve the degenerated phenotype of human IVD cells. De-differerentiated human IVD cells and DWJM were combined in a 3D culture condition without differentiating agents, cytokines or growth factors, showing that the mere presence of the matrix positively affected the expression of critical regulators of IVD homeostasis, such as SOX2, SOX9, and TRPS1 (Penolazzi et al., 2019).

Wharton’s jelly from umbilical cord is becoming increasingly proposed as an ideal source for obtaining scaffold useful for tissue engineering application (Beiki et al., 2017; Basiri et al., 2019). WJ is easy to obtain as biological waste material, is non-immunogenic, and its use presents no ethical concerns (Forraz and McGuckin, 2011). WJ contains abundant collagenic structural proteins (collagen I, III, VI, and XII), other ECM components such as fibronectin, lumican, heparin sulfate, and growth factors (TGFβ, FGFβ, VEGF) (Jadalannagari et al., 2017). It has been demonstrated that DWJM is able to retain many chemotactic factors and soluble bioactive factors positively affecting cell viability and function (Basiri et al., 2019). Therefore, for these different reasons DWJM has been proposed as potential therapeutic agent in many areas (Jadalannagari et al., 2017; Kehtari et al., 2019; Li et al., 2019). A still unexplored field is represented by the degenerated IVD. For the first time, in the current study we cultured human de-differentiated IVD cells from degenerated IVD in combination with DWJM. The attempt to restore the properties of the IVD cells through the peculiar characteristics of the DWJM, meets the need to develop alternative methods to the use of cell-based therapy that in many cases proved to be a failure (Sakai and Andersson, 2015). It has been in fact demonstrated that degradation and loss of ECM proteins associated with the upregulation of proteinases, such as ADAMTSs and MMPs, and downregulation of TIMPs (Vo et al., 2013), in the degenerated IVD produces a physicochemical microenvironment which is hostile for the engraftment of many types of transplanted cells such as MSCs from bone marrow or stromal adipose derived cells, compromising the cell-based therapy outcome in different clinical trials (Tendulkar et al., 2019).

The benefit derived from the presence of the DWJM is demonstrated by evidence we obtained and is distinguishable in two different phases. In a first phase, corresponding to the first seven days in culture, the presence of SOX2, a well known stemness regulator, and SOX9, the primary driver during the early stages of chondrogenesis, has been appreciably highlighted. The progressive increase of SOX2 and SOX9 expression levels in the first seven days of culture, suggests a functional cellular activity both in terms of stemness and differentiation supported by the presence of DWJM. Otherwise, the expression of SOX2 and SOX9 in degenerated IVD cells in culture was very low or undetectable as by us previously demonstrated (Penolazzi et al., 2019). It is noteworthy, that, regarding in particular SOX9, there is a growing interest on therapeutic approach based on protein targeting, by adenoviral vector (AdSOX9) intradiscal injection aimed at increasing the percentage of positive resident cells (Paul et al., 2003), or by delivery of SOX9-transduced MSCs (Sun et al., 2014). In a second phase, between day 7 and day 14, IVD cells combined with DWJM showed a down-regulation of SOX2 and SOX9 together with an up-regulation of TRPS1, a chondrogenic transcription factor previously identified by us as chondroprotective and associated with the lower grade of disc degeneration (Penolazzi et al., 2019).

Our data are in line with the hypothesis that the strengthening of cell properties in terms of viability and expression of specific proteins at precise times represents an important condition in the perspective of guiding the recovery of cellular functionality and triggering regenerative potential. Therefore, the potential of DWJM to revert degenerated IVD cells can be exploited to carry out an ECM-based intradiscal injectable therapeutic (Tukmachev et al., 2016; Wachs et al., 2017; Porzionato et al., 2018; Zhou et al., 2018). In other words, the presence of DWJM could be sufficient to further the functional recovery of the endogenous cells in the degenerated IVD microenvironment. Otherwise, when MSCs were combined with DWJM, the main effect was a significant increase in cell proliferation, suggesting that these cells reseeded in the ECM they produced, just maintain their primitive phenotype without moving toward discogenic differentiation.

Moreover, it is worth considering also important technical aspects that arise from the data collected in the current study: i. producing an informative 3D culture system based on DWJM even with a low number of cells like those that can be obtained from a human IVD biopsy; ii. providing a cell culture – based platform available as a preclinical experimental model which can be exposed to different treatments mimicking pathophysiological IVD microenvironment (hypoxia, dynamic culture condition, mechanical forces), as an alternative to animal models such as rodents, rabbit or other large quadrupeds (Alini et al., 2008); iii. making available an ECM – based product characterized not only by biological properties suitable for cell survival and function, but also easy of handling for clinical application. DWJM can be easily stored as lyophilized powder, and also modified as ready-to use injectable hydrogel treatment for intraoperative application (Tukmachev et al., 2016; Wachs et al., 2017; Porzionato et al., 2018; Zhou et al., 2018).

Finally, we should also consider some limitations of this study as well as further investigation to demonstrate the potential of DWJM employment in restoring IVD integrity and functionality. First, we demonstrated the maintenance of ECM microarchitecture after decellularization process, however, further analysis are needed to define the relationship between ECM and IVD cells, and cell-cell interactions. In particular, being cell adhesion a critical event affecting cellular signaling, survival, growth, and phenotype, cell surface receptors such as integrin subunits, distribution of fibronectin, laminin and collagen receptors, the involvement of Focal Adhesion Kinase (FAK) and Integrin-linked Kinase (ILK) pathways (Cukierman et al., 2002) deserve further study. This aspect could help to better understand IVD cell differentiation mechanisms. Second, there are some recent studies that have focused on the effect of decellularized IVD ECM on treating disc degeneration in animal models (Lin et al., 2016; Illien-Jünger et al., 2016; Zhou et al., 2018). It would be interesting to compare the properties of this matrix with DWJM in order to optimize specific tissue engineering applications. Moreover, SEM and TEM microscopy revealed the presence of numerous extracellular vesicles (EVs) in DWJM combined with IVD cells. It remains to investigate whether the effect of DWJM on improving the IVD cell phenotype should be attributed to the 3D environment itself, to the native WJ microenvironment and the bioactive molecules of a rich ECM that are preserved after decellularization, or to a specific paracrine activity of the IVD cells triggered by the DWJM itself. Recent evidence suggests that the production of EVs often happens when cells are combined with collagen based biomaterials (McNeill et al., 2019). It will be particularly interesting to understand how DWJM is able to induce EVs production when combined with IVD cells but not with MSCs from Wharton’s jelly. Therefore, in order to improve the comprehension of the mechanism underlying the action supported by DWJM, and to develop innovative therapeutics for spine injuries based on single molecules, in the next future it will be useful to analyze also EVs protein content.

## Data Availability Statement

All datasets generated for this study are included in the article/Supplementary Material.

## Ethics Statement

The studies involving human participants were reviewed and approved by the Ethics Committee of the University of Ferrara and S. Anna Hospital. The patients/participants provided their written informed consent to participate in this study.

## Author Contributions

LP, MP, EL, and RP: conceptualization, experimental design, and writing. LP, LB, SD’A, and RF: methodology and visualization. GB, PD, and MC: supervision and data collection.

## Conflict of Interest

The authors declare that the research was conducted in the absence of any commercial or financial relationships that could be construed as a potential conflict of interest.
