# Supplementary figures and images for: Extracellular Matrix From Decellularized Wharton’s Jelly Improves the Behavior of Cells From Degenerated Intervertebral Disc
Source: Front Bioeng Biotechnol. 2020 Mar 27;8:262. doi: 10.3389/fbioe.2020.00262 (PMC7118204; doi:10.3389/fbioe.2020.00262)

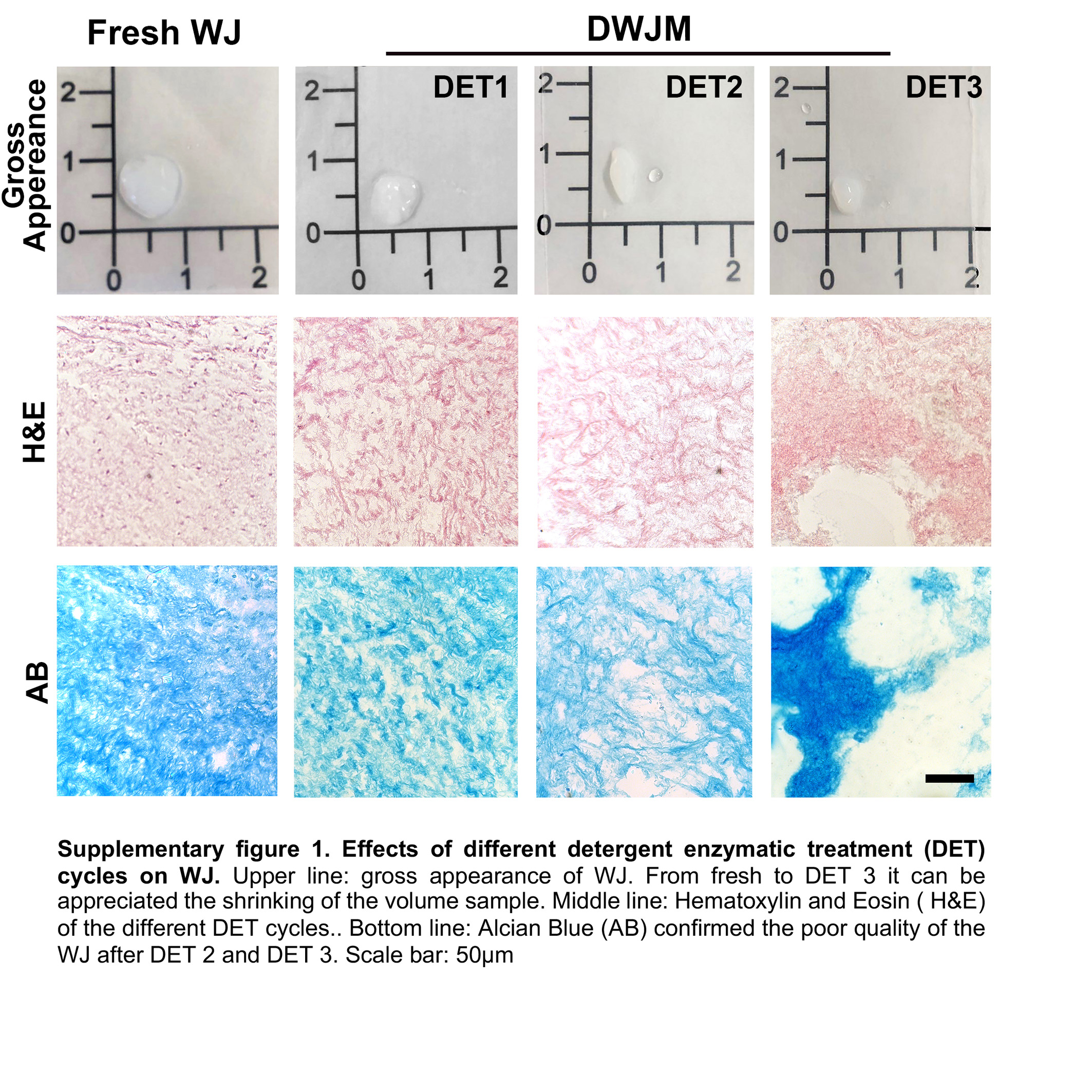

Supplement: Supplementary file 1 [file Image_1.JPEG]

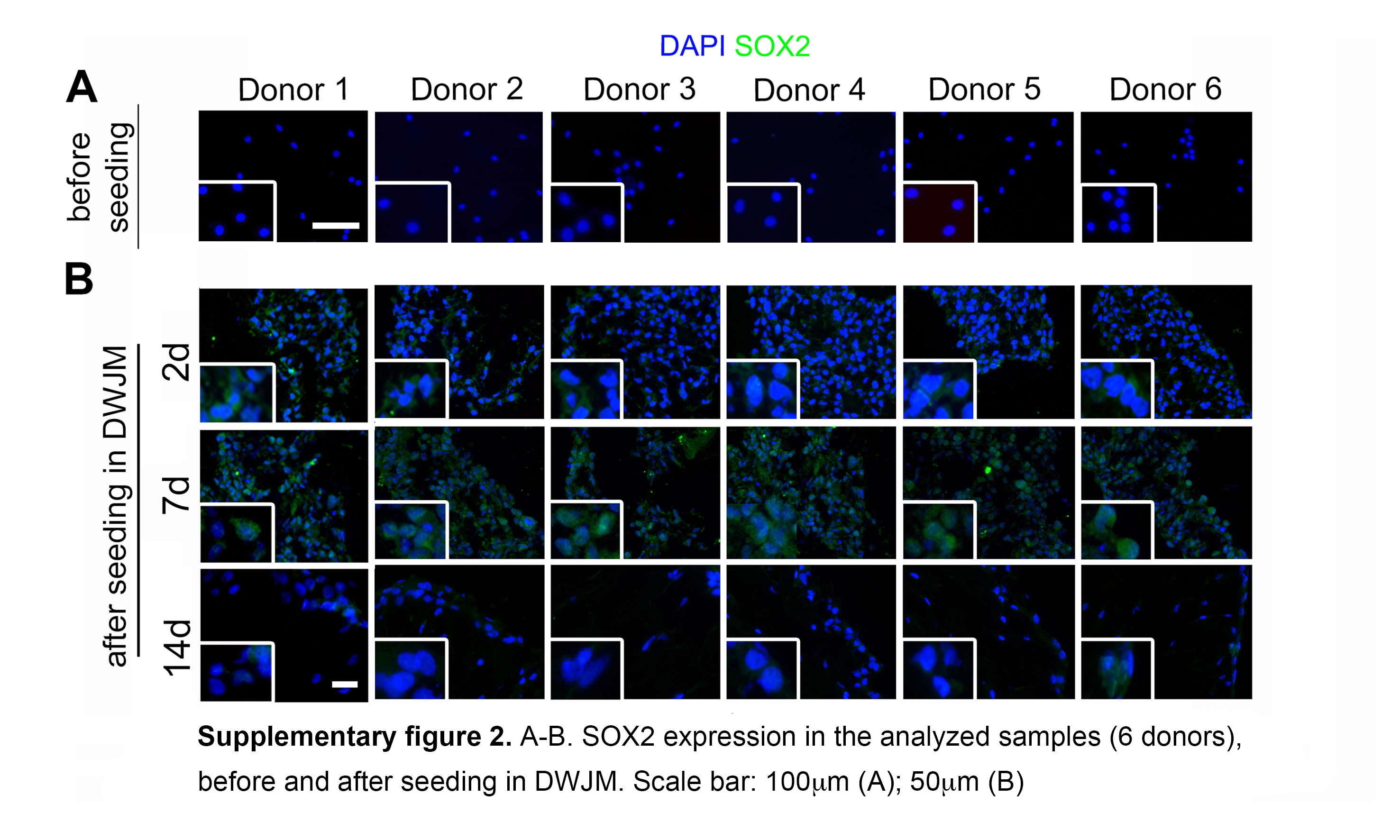

Supplement: Supplementary file 2 [file Image_2.JPEG]

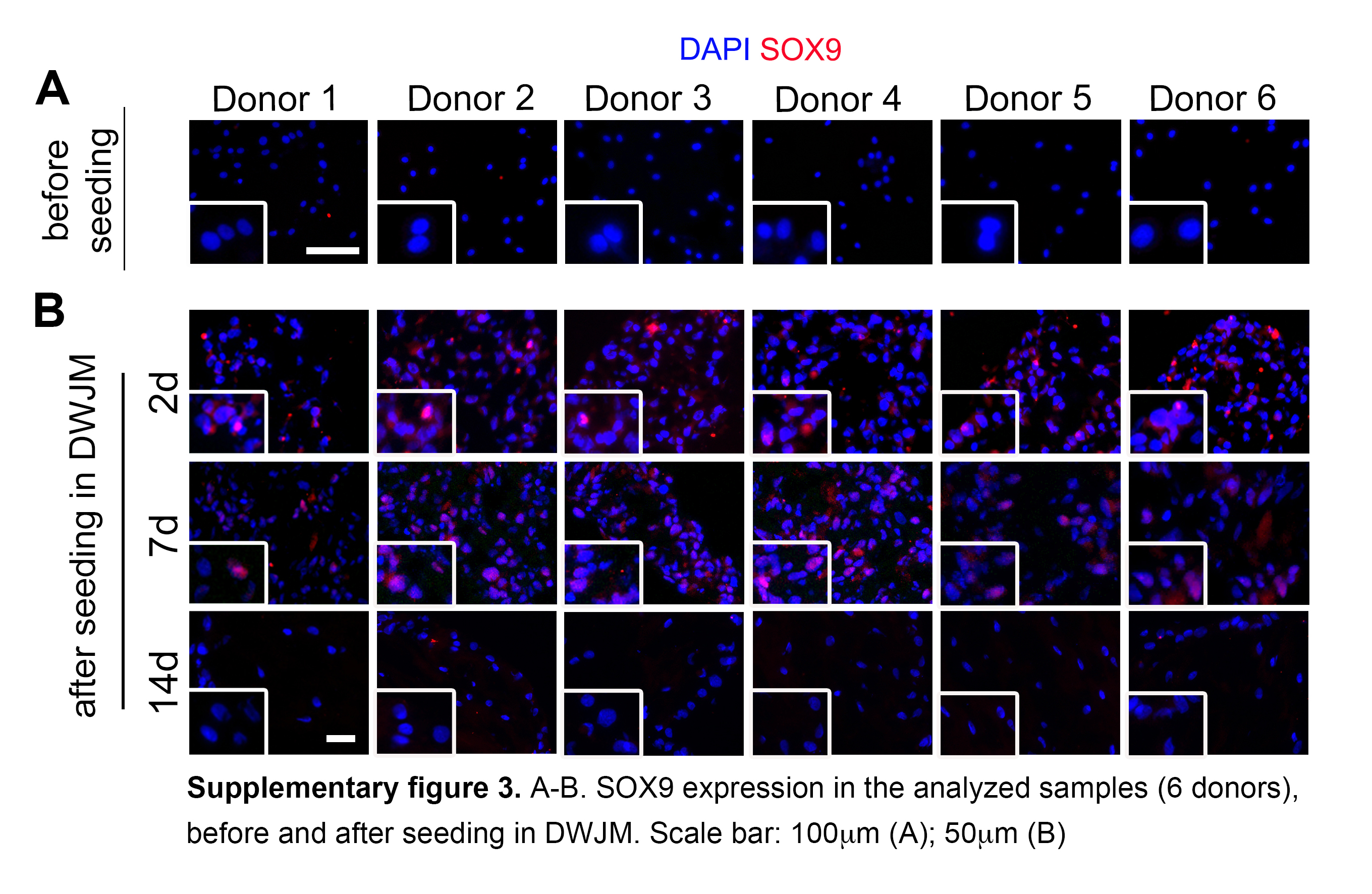

Supplement: Supplementary file 3 [file Image_3.JPEG]

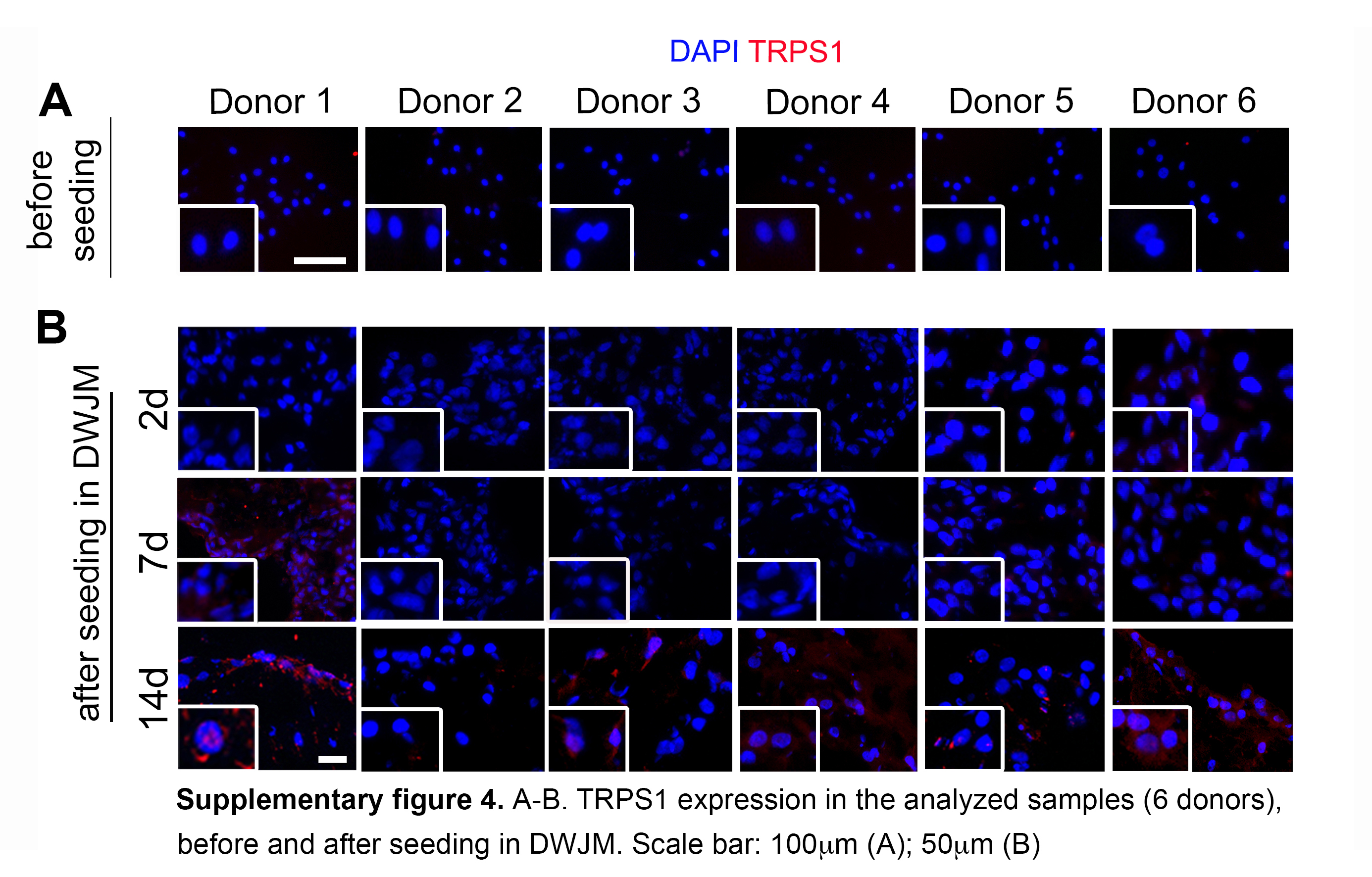

Supplement: Supplementary file 4 [file Image_4.JPEG]
