# Supplementary material for: Extracellular Matrix From Decellularized Wharton’s Jelly Improves the Behavior of Cells From Degenerated Intervertebral Disc
Source: Front Bioeng Biotechnol. 2020 Mar 27;8:262. doi: 10.3389/fbioe.2020.00262 (PMC7118204; doi:10.3389/fbioe.2020.00262)
Supplement: Supplementary file 5 [file Table_1.DOCX]

| ***Antibody*** | ***Dilution*** | ***Incubation time and temperature*** |
| --- | --- | --- |
| Mouse anti-human SOX2 (sc-365823), *Santa Cruz Biotechnology, Dallas, TX, USA* | 1:100 | 1 hour at 37°C |
| Rabbit anti-human SOX9 (sc-20095), *Santa Cruz Biotechnology, Dallas, TX, USA* | 1:100 | 1 hour at 37°C |
| Rabbit anti-human TRPS1 (20003-1-AP), *Proteintech Group, Rosemont,WA, USA* | 1:80 | 1 hour at 37°C |
| Goat anti-Mouse IgG Secondary Antibody, Alexa Fluor 594, *Invitrogen, Milan, Italy* | 1:200 | 1 hour at 37°C |
| Chicken anti-Rabbit IgG Secondary Antibody, Alexa Fluor 488, *Invitrogen, Milan, Italy* | 1:200 | 1 hour at 37°C |

Supplemental Table 1. Primary and secondary antibody list
